# Gambling, fast food and alcohol sponsorship in elite sport – perspectives from Australian sporting fans

**DOI:** 10.1186/s12889-022-14479-w

**Published:** 2022-11-23

**Authors:** Tara Boelsen-Robinson, Anne-Marie Thow, Nancy Lee, Tim Gill, Stephen Colagiuri

**Affiliations:** 1 Charles Perkins Centre, Faculty of Medicine and Health, Boden Initiative, University of Sydney, Sydney, 2006 NSW Australia; 2 Menzies Centre for Health Policy and Economics, School of Public Health, University of Sydney, Camperdown, NSW Australia; 3 Global Obesity Centre, Deakin University, Institute for Health Transformation, Geelong, VIC Australia; 4 Charles Perkins Centre, University of Sydney, Sydney, 2006 NSW Australia

**Keywords:** Sport, Sponsorship, Marketing, Qualitative, Child, Commercial determinants

## Abstract

**Background:**

Public health bodies in Australia remain concerned about marketing of unhealthy commodities; namely unhealthy food, alcohol and gambling products. Children are particularly susceptible to the influence of unhealthy commodity marketing. This study explored adults’ perceptions of unhealthy commodities sponsorship in elite sport and policies to restrict them.

**Methods:**

Four focus groups of 7–8 frequent sport spectators were recruited, including parents and non-parents, and located in inner and outer suburbs of Sydney, Australia. Results were analysed thematically.

**Results:**

Participants identified the contradictions of healthy messages of sport and unhealthy commodities, while highlighting the commercial value of sport sponsorship to sporting clubs. There is concern around children’s exposure to effective and integrated marketing techniques when viewing sport, which encouraged unhealthy habits. Support for restricting sponsorship related to perceived product harm, with gambling viewed as having the greatest health impact. Participants were supportive of policies that reduced exposure of unhealthy commodities to children, but were concerned about the financial risk to sporting clubs. Governments and sports associations were identified as holding responsibility for enacting changes.

**Conclusion:**

A number of options were identified for advocates to gain public and political traction to reduce unhealthy commodity sponsorship. There is potential for shifts away from unhealthy sponsorship by both governments and sports associations.

**Supplementary Information:**

The online version contains supplementary material available at 10.1186/s12889-022-14479-w.

## Background

Corporate marketing of products and services is a key activity which influences and impacts health outcomes [1]. Marketing is an effective means to persuade individuals to purchase products and services, including those that can be detrimental to health such as alcohol, gambling, and unhealthy foods and beverages (those high in saturated fats, added sugars and/or salt[2]). Children are particularly susceptible to the influence of marketing [3, 4]. Food marketing is effective at directing children’s preferences, choice and consumption of those products [5, 6], while exposure to alcohol marketing has been linked to hazardous alcohol consumption in young people [7]. Marketing of gambling products triggers impulses to gamble amongst problem gamblers [8] and creates gambling intentions amongst children [9].

The World Health Organization (WHO) has made formal recommendations to protect and promote health through reducing exposure to, and power of, harmful marketing practices of unhealthy foods and beverages [10], alcohol [11] and gambling [12]. These ‘unhealthy commodities’ have been the focus of recommendations by public health experts and advocacy groups, who stress the importance of having comprehensive regulations that include all forms of marketing that children may be exposed to (including but not limited to – television, digital media, sport sponsorship, branding, and giveaways) [13, 14]. In Australia, the combination of legislation and self-regulated standards has been criticized as failing to protect children from exposure to a range of unhealthy commodity marketing across a broad spectrum of mediums and marketing methods [15–17]. As well as these broad issues, there has been particular focus on a number of mediums [18–20], one of which is brand sponsorship of elite sport [21].

Sport sponsorship forms part of a strategic marketing approach to increase sales, enhance corporate image and build brand awareness by associating the positive attributes of and feelings towards, a sport, an event, or specific team with the sponsoring brand [21–24]. Australian sport spectators are exposed to marketing practices promoting unhealthy food, alcohol and gambling brands and products through multiple types of promotion [25–29], as are international audiences [30, 31]. A 2015 Australian study found that 27% of brands sponsoring cricket were alcohol, gambling or unhealthy food and drink products [32]. Children are frequently able to identify which junk food, alcohol and gambling brands are associated with different sporting teams [33], while exposure to unhealthy food sponsorship increases favourable attitudes to brands in young adults [34] and increases the awareness, appeal, and purchases of endorsed brands in children [35]. In recognition of the potential harm of sport sponsorship, there have been targeted calls for elite sport to restrict sponsorship by brands that represent health-harming products both in Australia [36, 37] and abroad [38, 39]. This follows the successful restriction of marketing of tobacco brands and products via sports sponsorship in Australia and elsewhere [40–42]. A particularly successful element of this strategy in Australia was the government buy-out of tobacco sport sponsorship using funds from tobacco taxes [40].

Australians are generally concerned about the impact of unhealthy marketing practices on children and there is public support for a range of broad marketing and sport sponsorship-focused restrictions related to these products. Most research to date has examined attitudes towards restrictions that protect children from unhealthy food marketing, with support for restrictions ranging from 58.4 to 87.0% in several recent, representative surveys [43–46]. Two further studies reported that between 40 and 44% of Australians are supportive of restricting unhealthy food brand associations with sporting events [46, 47]. Australians also appear to be supportive of greater bans on the advertising [43] and marketing of alcohol [48]. Similarly, around 80% of surveyed Australian’s were supportive of restricting gambling advertising during televised sport and banning advertisements for gambling from sporting venues [49].

However, individuals hold complex views and narratives regarding sports sponsorship by brands representing unhealthy commodities. These go beyond simply supporting or opposing policy change, and understanding these on a deeper level may be helpful for framing messages and garnering support for public health policy measures [43, 50]. Focus groups are an effective means to deeply explore issues and perspectives related to public health policies [51, 52], with recent calls to increase qualitative exploration of the acceptability of prevention policies [53]. Qualitative explorations of support for marketing restrictions in Australia have focused on sponsorship of non-elite sport [54], television [55], supermarkets [56], or broader explorations of government policies for preventive health [43] or nutrition [57]. A qualitative exploration of public opinion related to the marketing of unhealthy commodities through sports sponsorship and policies to restrict them has not been conducted in Australia, the results of which can inform advocacy efforts and policy development. Further, reflections from the decades-long fight to restrict and remove tobacco advertising within Australia highlight the need to create fresh perspectives and narratives in order to maintain or re-engage media, public and political interest in the topic [40].

In this study, we aimed to explore people’s perceptions of sponsorship of unhealthy food, alcohol and gambling brands and products (‘unhealthy commodities’) in elite sport. In addition, we asked about the acceptability of policy options to restrict unhealthy commodities in sport, how participants framed their support and opposition to these policies, and who they viewed as being responsible for enacting them.

## Methods

### Overall approach

A qualitative research design was employed using focus groups to capture participants’ perspectives and allow them to reflect on and engage with the opinions of others [58]. Four focus groups were conducted, each with 7–8 participants. These numbers were expected to produce data of sufficient depth and richness to achieve data saturation and answer the research questions [59–61]. Recruitment was conducted using an external market research company, Farron Research (further details of recruitment in Supplement 1). Focus groups were conducted in professional focus group facilities with one-way mirrors and were audio and video-recorded. NL and TBR alternated between moderating and observing the focus groups while taking field notes. Participants were reimbursed for travel and participation time at standard rates. Focus groups were thematically analysed. Ethics approval was granted by the University of Sydney (protocol number 2019/726).

### Discussion guide development

Four exploratory pilot focus groups were conducted to explore consumers’ perception of the role and potential harm of marketing in different forms, and amenability to different forms of marketing regulation (see Supplement 1 for further details). Sport sponsorship by unhealthy commodities (including unhealthy food and drinks, alcohol, and gambling) arose as a key area of concern, and guided the development of the discussion guides for the subsequent focus groups reported here, to explore perceptions, concerns with and solution to, elite sport sponsorship of unhealthy commodities - unhealthy food and drinks, alcohol, and gambling (see Supplement 2). Discussion guides were refined by consultation with experts to identify topics of interest, contention, and where further evidence was required in order to increase the salience and relevance of the study findings to the current policy landscape.

### Recruitment

We recruited participants who self-identified as frequent viewers of popular elite sport at an Australian and international level (viewing cricket, tennis, Australian Rules Football-AFL, Rugby League, Rugby Union, and/or soccer at least once per week), to allow them to reflect on their own interactions with, and perspectives of, elite sport sponsorship. Participants were grouped on the basis of their parental status, in order to identify any thematic variation or consistency in responses between and within these groups. Final focus groups were: two groups living with at least one child aged 3–12 years (one group conducted in central Sydney (FG1), and one in the outer suburbs in Sydney to allow for greater spread of participants (FG4)), one group living with at least one adolescent aged 13–17 years (conducted in central Sydney, (FG3)), and one group not living with children or adolescents (conducted in central Sydney, (FG4)). Participants were recruited to obtain a spread of characteristics of gender, socio-economic position (SEP) and cultural and linguistic diversity. Focus groups were conducted over 60 min.

### Analysis

The lead author (TBR) conducted a four part thematic analysis process as outlined by Green et al. [62]; immersion in data, coding, category creation and theme identification. A block and segment approach to coding was employed, where descriptive codes were applied to sections of text [63]. Codes were deductively identified based on the discussion guide sections and questions inductively identified as arising from the text. Codes were organized into broad categories of similar concepts, and themes identified through interpretation of the codes and categories in light of the research questions and arising from the text. Themes arising across and between the different focus groups were compared and contrasted, and were refined through discussion between the research team.

## Results

Twenty-nine individuals attended the focus groups, with 7–8 participants in each group. A description of participants is provided in Supplement 3.

Thematic analysis identified that support for restricting unhealthy commodity sponsorship was strongest for the brands viewed as most nefarious (gambling), and for measures which reduced marketing exposure to vulnerable groups (children). Responsibility of enacting such changes was seen to lie with governments and sports associations. A summary of themes emerging from focus groups are provided in Table 1. There were small discernible differences in focus groups, which are discussed within each theme. Quotes are used to illustrate commonly arising narratives and themes, and further examples are available in Supplement 4.

**Table 1:** Summary of thematic analysis of focus groups

Theme	Sub-theme	Description
Sporting experiences	Personal experiences	Sport evoked feelings of pride, nostalgia, and competitiveness, and was closely tied to community, family and friends.
Ubiquity and pervasiveness of marketing	Increased marketing integration and effectiveness	Due to sophisticated marketing techniques using integration and technology, marketing is more persuasive now than ever before.
Role of unhealthy commodities in sport	Commercial benefit of sponsorship	Businesses engaged in sport sponsorship to sell products, build brand reputations and associate with the positive attributes of sport
	Effectiveness of marketing and sponsorship	While individuals rarely noted their own susceptibility to marketing, certain groups such as children were more readily influenced.
	Contradictions of unhealthy commodities and sport	Deep and inherent contradictions between the healthy messages of sport and the potential negative health outcomes of fast food, alcohol and betting.
	Unhealthy habits associated with viewing sport	Consuming fast food, alcohol, and placing bets were all common experiences associated with or around watching elite sport.
Perceptions and opinions on restricting sports sponsorship and sport-related marketing	Mixed support	The appetite for restricting unhealthy commodity sponsorship depended on the perceived harm, exposure to children, marketing techniques used, and the ability of sports clubs to survive.
	Reduce exposure over complete restrictions	For alcohol and fast food, reducing exposure of marketing was preferred over complete restrictions, while there was more support for removing gambling from sport altogether.
	Survival of sport	Opposition to reducing unhealthy commodity sponsorship often came from concern that elite sport would lose income and become unviable.
Rights and responsibilities	Joint responsibility	Governments had the responsibility to protect children from harmful products, sports associations should act in the best interests of the community, and companies should not market harmful products where children will be exposed to them.
	Complementary approaches	Reducing exposure of marketing through sport sponsorship should be combined with other approaches to support healthy choices.
	Visible policy coherence	Governments should act on sport sponsorship to be in line with their other messages and actions on unhealthy eating, alcohol and gambling.

### Sporting experiences

Participants watched a wide variety of sport, including tennis, basketball, Australian Football League (AFL), soccer, netball, cricket, rugby union and National Rugby League (NRL) with their families, friends, and by themselves, in a variety of settings and through different mediums. Participants frequently noticed billboards, banners, advertisements shown during televised matches, and tie-ins with brands that were incorporated into the sporting match.

### Ubiquity and pervasiveness of marketing

#### Increased marketing integration and effectiveness

Respondents conveyed that exposure to branding and marketing was overwhelming and inevitable within their daily lives and believed that even if sport sponsorship and sport-related marketing (i.e. advertisements during sporting matches) was reduced, both children and the general population would still be bombarded with unhealthy commodity marketing. The influence and potential danger of marketing had increased over time, which participants related to increased exposure and more sophisticated, integrated, and persuasive marketing techniques.

*‘…it’s just so insidious, when you’re watching the game, compared to when I grew up watching rugby league, like they didn’t say anything about gambling. And now it’s like, it comes up… and showing the odds changing and things like constantly updating, and making that seem exciting as well. I just think it shouldn’t be included, full stop.’ Focus group 4, Person 3 (FG4, P3)*.

Marketing techniques had become more persuasive over time, due to technology (i.e. mobile applications) enabling people to immediately act to purchase food or gamble, and the integration of advertisements in game play. The greater the persuasiveness of the type of marketing strategies used, the higher participants perception of its harm. Participants also noted that they were more aware of the dangers of unhealthy commodities due to social marketing campaigns.

### Role of unhealthy commodities in elite sport

#### Commercial benefit of sponsorship

Participants perceived businesses as engaging in marketing activities for short- or long-term commercial benefits, such as increasing product sales, building brand reputation, tax breaks, or stakeholder management. The commercial benefits sought by the brands were perceived as differing, depending on the product – participants identified that fast food and gambling advertisements were aimed at producing immediate purchases, while banks and airline brands aimed to build positive reputations, particularly in associating the positive attributes of sport with the brand in question.

*‘With the fast food, they might get a quick sale out of it but when it comes to the bigger banks and stuff like that, I think it’s more reputational for them.’ FG2, P5*.

#### Effectiveness of marketing and sponsorship

Reflecting on their own susceptibility to marketing, participants largely believed that while they were immune themselves, others would be influenced. Respondents viewed children, people from disadvantaged backgrounds, with addictive personalities, or those already considering changing brands would be more responsive to marketing and sponsorship, with children being particularly susceptible to sporting stars promoting unhealthy products. Participants recalled specific instances where their children had been exposed to marketing and sponsorship of unhealthy commodities, which in turn they had convinced their parent to purchase through pester power.

*‘For me it’s the kids, if I’m watching with the kids, and they say*, “Daddy, can we have McDonald’s?” *because I’m excited, yeah, yeah, yeah, maybe* [I’ll] *say we buy McDonald’s.’ FG1, P3*.

Parents were particularly concerned with their own children’s exposure to gambling and betting advertisements through elite sport sponsorship, and also viewed fast food sponsorship in junior sport of particular concern due to the association of unhealthy food with a healthy activity like sport.

#### Contradictions of unhealthy commodities and sport

The benefits of sport, both physically and mentally, were perceived as being in direct opposition to the consumption of fast food, alcohol and gambling. Participants focused on the incongruity between the values and positive attributes that sport promoted and unhealthy fast food, with obesity, childhood obesity and cardiovascular disease as long-term outcomes of excessive fast food consumption.

*‘I think it [*sport sponsorship by unhealthy commodities] *defeats the purpose of what sport is all about, isn’t it? Again, like what I said before, it’s associated with health, with all the positive sides … And we all know that gambling, alcohol and fast food …more often than not, they have negative effects on our health so they’re actually on the polar sides of the health spectrum, if we want to promote healthy lives and healthy minds.’* FG2, P7.

While fast food was seen to be the most oppositional to the values promoted by sport and physical activity, alcohol and gambling were of higher concern to most focus groups due to the greater perceived harm of these products (differences explored below). Participants’ strength of support for restricting sport sponsorship tended to mirror the perceived harm of the product, with gambling consistently identified as the most nefarious product, resulting in devastating personal and community financial and mental health consequences. Close ties with unhealthy commodities were seen to reflect negatively on the sport itself. There was concern that the association with sport would imbue the unhealthy commodities with a misleading perception of healthiness (aka a ‘health halo’), making them more attractive to consumers. Gambling, alcohol and fast food were sometimes referred to as ‘addictive’ products by participants when supporting marketing restrictions of these products. Respondents agreed that alcohol consumption and exposure to alcohol marketing was harmful for children, were less clearly articulated than the harms of fast food and gambling.

FG2 (parents of adolescents, central Sydney) displayed a particularly strong aversion to all unhealthy brands sponsoring sport, and subsequent strong support for restrictions (explored below). In contrast, FG1 (parents of children, central Sydney) focused on the contradictions of fast food sponsorship in sport, with fewer mentions of alcohol and gambling sponsorship. While gambling was generally viewed as nefarious, FG2 (parents of adolescents, central Sydney) and FG4 (parents of children, outer suburbs Sydney) were particularly averse to children’s exposure to marketing of gambling products. FG4 were also concerned with the health impacts of energy drinks in children, however this issue was not raised by other groups.

#### Unhealthy habits associated with viewing sport

Respondents were consistently of the opinion that sport sponsorship was likely to have very little additional influence on their own actions given their existing strong habits surrounding consumption during sport viewing. Participants frequently consumed alcohol, fast food, and gambling surrounding or during viewing elite sport, across a number of different viewing contexts. Indeed, respondents understood the consumption of unhealthy commodities to go hand-in hand with viewing sport, as part of the ritual of watching sport. This perspective was despite strong agreement that unhealthy commodities were at odds with the health-promoting messages of sport and physical activity.

*‘And the only reason they don’t ban alcohol is because they see sport as not just being fit and healthy, it’s a cultural thing and drinking while watching it.’ FG2, P2*.

### Perceptions and opinions on restricting sports sponsorship and sport-related marketing

#### Mixed support

Participants displayed mixed support for restricting sponsorship of unhealthy commodity brands from sport altogether, with the strength of support for more stringent measures coalescing around four factors: (1) the perceived harm to individuals and society of the product in question; (2) the potential exposure to children; (3) the perceived persuasiveness of the type of sponsorship or marketing technique; and (4) whether the sport or sporting club would be able to survive financially.

*‘I think depending on how they do it [restrict sport sponsorship]. If they limit by time during which you can advertise or something, I think that would probably be welcomed by most of the public.’ FG3, P2*.

FG2, in line with their perceived harm of sport sponsorship of unhealthy commodities, demonstrated the strongest support for removing all unhealthy commodities in sport. FG1 participants were noticeably less supportive of action on sport sponsorship, citing instead that parental responsibility was key in reducing the exposure of children to unhealthy commodity marketing. FG1 were all parents of children, but their views were in contrast to the other parents of children, FG4, who did not share this belief to the same extent.

#### Reduce exposure over complete restrictions

While some participants demonstrated strong views on restricting all forms of unhealthy commodity sponsorship in sport, there was general agreement about the importance of reducing children’s exposure to unhealthy marketing. Respondents viewed gambling and betting as the most harmful products to individuals, followed by alcohol and fast food, which paralleled the support for restricting sponsorship by brands selling these products. While there was no differentiation between the types of gambling and alcohol products marketed, participants identified that fast food brands could sell both healthy and unhealthy options. Thus, there was more support for restricting the marketing of ‘unhealthy’ products compared to brands as a whole.

When participants discussed solutions to sport sponsorship, they often focused on the type of marketing they viewed most persuasive and thus harmful – television advertising viewed during sport, rather than other forms of sport sponsorship such as branding on jersey, or signage at matches. There was mixed responses to the personal importance of stopping unhealthy sport sponsorship; for some it was essential, but others noted that while it may be important for others and the community, it was not for them.

#### Survival of sport

Respondents frequently drew parallels with the banning of tobacco advertisements and sponsorship in sport. Participants were highly receptive to the idea of the government ‘buying out’ unhealthy product sponsorship – where funded health promotion messaging replaces targeted options (when explained to them by the researchers), similar to the approach taken towards tobacco products with the caveat that it wouldn’t take away from frontline healthcare funding. Participants identified that sporting teams, and broadcasters, were reliant on income from sponsorship and marketing deals to be viable.

*‘I think with the money that comes in from advertising on that, and then we want free to air networks to still hold sport, earn money, for the amount of money it costs them to win the rights, that if you ban another thing that advertises quite regularly, then you might be kissing your free to air TV goodbye pretty quickly.’* FG1, P4.

Participants noted that sports teams were able to overcome the removal of tobacco sponsorship, but had mixed views as to whether they would be able to continue if gambling, alcohol and fast food sponsorships were to be restricted. Respondents were concerned about ‘where to draw the line’. As stated above, participants resolved these tensions by preferencing approaches which limited the exposure of children to unhealthy commodity marketing over restrictions on whole brands or companies. However, participants view of gambling as extremely harmful underpinned their support for the complete removal and dissociation of this product from elite sport.

### Rights and responsibilities

#### Joint responsibility

Participants held the perspective that sports associations, governments television networks, consumers and companies had a joint responsibility to take care of the community. Government and sports associations were perceived to have the main responsibility to reduce unhealthy commodity sport sponsorship. Governments were viewed as needing to take responsibility to protect the health of the population through regulation, however, some respondents questioned their ability to implement such restrictions in practice.

*‘I think we are quite firm in our minds that we don’t enjoy fast food, we don’t enjoy gambling, so it’s not so much for us but more the broader societal impact of it. Ideally, that would be restricted. Whether or not government can actually implement those restrictions without going too far is another question.’ FG3, P2*.

Participants saw the responsibility of sporting organisations to consider how their choices of sponsorship may impact the community. Respondents noted that being reliant on unhealthy commodities for sponsorship may pose a financial risk if government’s were to impose restrictions. Organisations were viewed as having a right to conduct marketing practices, however participants thought this right was limited when the marketed product caused sufficient harm, with gambling being a frequent example. Participants also noted they had expectations of organisations to act fairly by choosing to limit the exposure of children to their marketing of unhealthy commodities. In contrast, other participants were sceptical of companies selling unhealthy commodity to do the ‘right thing’, noting the financial gains of marketing to children. Respondents expressed a tension between a hesitancy to ‘overregulate’, and a desire to protect children who were viewed as requiring protection from influential marketing tactics.

*‘Again, these so-called ‘advertising ethics’ don’t exist because everyone knows the odds are heavily stacked.’ FG2, P3*

#### Complementary approaches

Respondents reflected on other means to reduce harmful behaviours – the role of individual responsibility, parents, and warning labels. Participants identified that individuals were able to choose to consume a product, despite the recognized influence of marketing. A divergent narrative emerged where parents were expected to protect their children from marketing of unhealthy commodities, while recognizing the inescapable nature of marketing exposure. Participants thought that governments could promote healthy lifestyles instead of banning unhealthy sport sponsorship.

#### Visible policy coherence

Government action in limiting sport sponsorship by unhealthy commodities would be consistent with government messages on health and other policy actions, as viewed by participants. Respondents identified that the current status quo of allowing sport sponsorship by unhealthy commodities (in particular by fast foods) was in contradiction to governments supporting healthy canteens and healthy eating lessons in schools, and in providing healthy activity vouchers for sport. Participants noted that the government had a financial stake in supporting the reduction of unhealthy commodity sponsorship due to the healthcare costs of unhealthy diets.

*‘To me, it’s a bit contradictory… they* [the government] *provide healthy recommendations for schools on their tuckshops and their shops in the schools – canteens – but then they allow things like advertising for fast food in sports and that and kids watch sport as well …’ FG2, P5*.

## Discussion

This is the first study to conduct an in-depth qualitative exploration of the acceptability of policies restricting sponsorship of elite sport in Australia by unhealthy commodity brands in Australia, and one of few internationally [35, 64]. Sport viewers were concerned about the harmful impacts of unhealthy commodity sport sponsorship and marketing. Our participants reflected on the incongruity and incompatibility of unhealthy food brand sponsorship in sport, a finding consistent with a previous Australian survey [47] and New Zealand focus groups [65]. In line with former studies, our participants displayed a strong aversion to gambling and betting in sport [66–68], as well as concern regarding the exposure, appeal and influence of these products on children [69]. Concern around unhealthy sponsorship of elite sport has been studied extensively in Australia, but there are indications that it is a concern of sports followers from across the world. A review by Ireland et al. (2019) identifies studies highlighting similar concerns with Turkey and the USA [70]. While participants were supportive of gambling restrictions, they were more cautious of supporting comprehensive policies to restrict sport sponsorship by unhealthy commodities more broadly. These results are in contrast to previous surveys indicating strong support for restricting unhealthy food and drink marketing in elite sport in Australia [45, 46, 71], which may be due to these studies using close-ended questions in a survey design, are reported independently, and focus on a broader population. Our focus groups, including only regular sport viewers, identified a more nuanced picture where support for restriction were related to higher perceived product harm and stronger effectiveness of marketing methods. Further, our participants may have been influenced by dominant voices in the group. Our findings that both parents and non-parents expressed similar levels of support for restrictions is similar to previous surveys exploring unhealthy food and beverage marketing restrictions [72].

Participants reflected on the integrated and persuasive nature of marketing, the strategic approach to sport sponsorship to influence purchases and brand perceptions, and the challenge of regulating unhealthy commodity marketing. These themes resonate with a growing body of academic literature [1] and public perceptions [57] recognising the importance of limiting the scale and power of corporate actions to address rising chronic diseases, conceptualised as the “commercial determinants of health”. Participants held strong views on the role of parents in protecting their children from harmful marketing practices and providing healthy options. This perspective is consistent with previous qualitative explorations [43, 57], and corporate messaging which emphasises personal responsibility in order to deflect from companies’ own influence. However, participants were also particularly concerned about the increasing exposure of children to sophisticated, targeted, and persuasive marketing interactions through digital and online platforms, within the context of viewing sport, and more broadly. This suggests that framing advocacy efforts to restrict unhealthy commodity marketing by highlighting the power and reach of corporations, and their integrated marketing approaches, and focusing on both commodities and mediums of concerns (gambling, digital technology, respectively), is likely to resonate with existing community perspectives and areas of concern.

The shared responsibility narrative that emerged from our focus groups corresponds with a previous Australian study examining preventive health policies [43] and highlights an opportunity to target both government and sports organisations as influential actors. Drawing on other examples of unhealthy commodity removal or reduction may be essential to building a credible argument for restrictions and reduce the sports clubs perceived risks of such actions. For example, Baseball Australia’s pledge to no longer accept alcohol sponsorship at a local or elite level [73, 74].

Participants expressed some concern with the potential financial impact of restricting sponsorship to elite sport, similar to previous findings from Australian sports administrators [54]. Public health advocates have identified a hypothecated tax as a solution to transition away from unhealthy commodity sponsorship, with the graduated removal of tobacco marketing (including sport sponsorship) in Australia represents an exemplar to follow [40]. The high level of sponsorship by unhealthy commodity companies and brands internationally [28, 75, 76] may represents a future financial risk to mitigate. Growing interests in health and nutrition amongst young people [77], their decreasing alcohol consumption [78], and the community’s deepening mistrust of gambling corporations abilities to act in good faith [79] means that removing or lessoning reliance on unhealthy commodity sponsorship may represent a safer long-term strategy.

Another potential framing which emerged from our focus groups was that restricting unhealthy commodity sponsorship is an opportunity for the government to increase its policy coherence in line with other healthy policies they promote and implement, such as healthy school canteens. The 2021 National Preventive Health Strategy identified both the marketing of unhealthy products, and access to digital platforms that deliver unhealthy products as causes of poor health [80]. There is an opportunity to bring these policies in line with changing societal expectations about the role of government in protecting populations against corporate interests, particularly around gambling as demonstrated by recent moves in the UK [74]. This approach draws strong parallels with the societal and political shift in opinion against tobacco companies and resulting policies restricting their marketing and sale [40]. Recent actions by global digital platforms, such as YouTube to allow users to limit exposure to gambling and alcohol advertising, reflect shifting expectations for corporations to limit harm despite conflict with their commercial goals [81]. The view of participants that fast food brands sell both healthy and unhealthy food products is seen by some researchers as a deliberate approach of food and alcohol companies to present a narrative that they are responding to consumers desires for healthier products, and thus reinforce the functioning of self-regulation and avoid the need for government intervention [82, 83].

### Implications for public health

This study identified a number of potential pathways for improved advocacy efforts on unhealthy commodity sponsorship in elite sport. Firstly, harnessing broad public support by focusing on community concern around the heightened exposure of children to gambling advertisements through sport, and the increasing access to unhealthy commodities through digital mediums which are integrated into sport sponsorship. A focus on children and impact of integrated digital mediums may also be appropriate avenues to advocate for broader marketing restrictions. Secondly, articulating to sporting bodies how they could successfully financially divest from unhealthy commodity sponsorship would mitigate a key concern. Advocates may also argue that a reliance on unhealthy commodity sponsorship is a reputational and financially risky strategy, which may position restricting or removal of sponsorship as a safer long-term alternative. Lastly, there is an expectation from the public that governments and sporting associations should be taking action in this space. Advocacy efforts should focus on communicating to policy makers on the societal expectations of governments to protect vulnerable populations from the harms of unmitigated corporate influence.

### Strengths and limitations

Our staged approach to collecting community opinions on commercial sponsorship of sport by unhealthy products has a range of strengths and limitations. The use of pilot focus groups to define the scope and guide the final focus groups enabled a clear focus on issues of community concern. Drawing our final focus groups sample from sporting fans means our findings are likely to represent that section of the community that would be most impacted and possibly resistant to changes to sport sponsorship. The use of four focus groups with 7–8 participants for the in-depth analysis provided the best opportunity to reach data saturation. However, we did not collect the personal behaviours of participants and were therefore unable to assess how their perspective aligned with personal behaviours such as gambling and consumption of unhealthy foods and alcohol. Our recruitment based on gender and socioeconomic position would have reflected a wide variety of viewpoints.

## Conclusion

This study identified new narratives and framing that may provide useful strategies for reinvigorating support for reducing unhealthy commodity sponsorship in elite sport. Effective advocacy should focus on highlighting children’s exposure to most harmful products (gambling), and most persuasive marketing methods (integrated, digital), as a means to advocate for broad restrictions of unhealthy commodities in sport. Allaying fears of financial risk by drawing on exemplars of best practice for governments and sports associations to follow are strategies with demonstrated effectiveness in parallel public health arenas.

## Electronic supplementary material

Below is the link to the electronic supplementary material.

Supplementary Material 1

## Data Availability

The datasets generated and/or analysed during the current study are not publicly available to protect research participant privacy but are available from the corresponding author on reasonable request.
