# Supplementary material for: Gambling, fast food and alcohol sponsorship in elite sport – perspectives from Australian sporting fans
Source: BMC Public Health. 2022 Nov 23;22:2155. doi: 10.1186/s12889-022-14479-w (PMC9685834; doi:10.1186/s12889-022-14479-w)
Supplement: Supplementary file 1 — Supplementary Material 1 [file 12889_2022_14479_MOESM1_ESM.docx]

Gambling, fast food and alcohol sponsorship in elite sport – perspectives from Australian sporting fans

**Supplementary material**

**Supplement 1: Pilot focus group**

## Methods

Recruitment was conducted using an external market research company, Farron Research which has a national panel of 200,000 individuals recruited via advertisements on Google and Facebook and screened via a questionnaire and phone interview. Potential participants were contacted by the recruitment company and screened and recruited using an online questionnaire according to the selection criteria detailed below until all groups were filled. NL and TBR alternated between moderating and observing the focus groups while taking field notes. NL is experienced in conducting focus groups and TBR has experience conducting one-on-one interviews. Participants were given an information sheet detailing the study and the contact details of the researchers during the recruitment process, and again immediately prior to the FG commencement. Participants were able ask the moderator questions regarding the study prior to signing the consent form and starting the study. Participants were reimbursed for travel and participation time at standard rates for focus groups.

Pilot focus groups 1-4 explored consumers’ perception of the role and potential harm of marketing, its impact on consumption, perspectives and amenability to different forms of marketing regulation, and whether these perspectives differed by type of product and form of marketing. The focus groups were exploratory in nature and particularly explored perspectives surrounding unhealthy food and beverage marketing. Participants were shown three advertisements for less healthy foods and drink products as a focal point for discussion to understand their perspectives on child-targeting, product content, and methods used in the ad. Participants were grouped based on the following characteristics; 1) Parents of young children; 2) Frequent free-to-air television viewers; 3) Frequent viewers of sport (live, free-to-air and/or paid) and; 4) frequent users of social media. Characteristics were based on current discourse surrounding unhealthy food and beverage marketing, recommendations for action, and specific criticisms of marketing regulations in Australia (1-8). Within each focus group, participants were recruited to obtain a spread of characteristics of: age (50% 18-35, 50% >35); gender (50% female); socioeconomic position (50% living in an area of low-socioeconomic position according to Socio-Economic Indexes For Areas, Index of Relative Socio-economic Disadvantage SEIFA IRSD (9)); and cultural and linguistic diversity (25% minimum to reflect the Australian population where 29.7% are born overseas (10)). Three focus groups were conducted in central Sydney, and one in the outer suburbs of Sydney. A descriptive analysis was conducted by the lead author (TBR) with input from all authors, which focused on identifying marketing avenues and products of concern, and policy solutions to reduce exposure of unhealthy food and beverage marketing to children. Sport sponsorship by unhealthy commodities (including unhealthy food and drinks, alcohol, and gambling) was consistently raised as an area of concern. In particular the inconsistency of fast food brands sponsoring sport, the volume of gambling advertisements showing on free-to-air television during sporting matches, and a focus on providing solutions for unhealthy commodity sponsorship in sport.

**Discussion guide Pilot Focus Groups**

**Discussion guide**

1. Introduction (5 mins)

Today we are going to discuss topics around marketing. We’re interested to know how marketing factors into your day to day life, especially around things that we consume in our day to day, the kinds of things we see marketed, and if we think certain things should or shouldn’t be marketed. There are no right or wrong answers. I also must let you know that we are being video- and audio recorded, and there are researchers sitting behind this mirror here. The ethics of market and social research dictate that if someone watching or listening to this group recognises one of you, they are obliged to leave the room. While I will ask for your names for our discussion, no one here will be explicitly named in this research.

**2. Warming up (10 mins)**

- Name, and one thing you enjoy doing for entertainment

**3. How and what media is consumed (10 mins)**

To make sure we’re all on the same page, I’m going to read out the definition of ‘marketing’ to you, because that’s what we’ll be talking about today: “activities undertaken by a company to promote the buying or selling of a product or service”.

- So, can you call out some examples of what you would consider marketing?
- What kinds of products or services are marketed? (NOTE any mentions of food or drink, probe on some examples)

Tell me about the kinds of media that are relevant to you

- - TV? Radio? Podcasts? Online gaming? What sites/apps do you use regularly?
- How do you use these media? i.e. do you watch TV in the living room with your family/housemates, do you stream in your room, do you look at your phone on the bus, etc.)
- How much time per day is taken up with TV? Social Media? Other apps?
- **IF PARENTS**: what about your kids? What do they like to use? How important is it to you to be aware of the media they’re looking at/using/reading? How do you talk to your kids about what media they consume?
  - Tell me about why it is or isn’t important to know what your kids are taking in. (**PROBE** on tools, if any, parents impart to kids about critical media consumption?)
- Thinking about the last 48 hours or so, what kind of goods and services did you see marketed? And where did you see it? (watched TV, played a computer game, used an app)?
  - What were they marketing? How? (note any mentions of food; if none come up after going around the table, ask – what about food or drink?)
  - What did you learn about the product and the way it was marketed? What appealed to you? What did not appeal?
  - How, in your opinion, can marketing be ‘effective’? Do you recall the last time you felt motivated to do something because of marketing? What was it? Why/how did it motivate you?

**4. Existing knowledge and opinions (10 mins)**

- What do we know about the rules around marketing – like what can and can’t be marketed?

There are certain things that the government regulates around marketing. For example, alcohol isn’t allowed to be marketed at children, and tobacco products aren’t allowed to be marketed at all.

- What do you think about that? What about freedom of speech? Should the government really be involved in private business (in terms of saying what a business can or can’t try to sell)? Do businesses have to be responsible about how they try to sell something? Isn’t it our own responsibility about what we do and don’t buy? Why? Why not? [Note if food comes up. If not, PROBE WITH EXAMPLES – cars, insurance, food]
- What about for children? What kinds of things shouldn’t be marketed to children? [See if there is unprompted mention of unhealthy food or beverage; note for further discussion later]
- We’ve covered what kinds of things, but what about where and how? What types of marketing and spaces should be off limits when we’re thinking about marketing? Are there any? What about marketing to children? Should anything be off limits? Whose responsibility is it, the companies doing the marketing? Parents?
- **[IF IT DIDN’T ALREADY COME UP UNPROMPTED]** What about unhealthy food and drinks?
- Let’s back up a little bit here – what do we mean by ‘unhealthy food and drinks’? Let’s make a list – what are ‘unhealthy’ foods? what *doesn’t count* as ‘unhealthy’ foods? What are ‘healthy’? [write on whiteboard] How do you think of these types of foods? Do you think, wow I feel like eating something unhealthy right now.
- Looking at these lists, can you tell me more about the items on these lists? What does the ‘unhealthy’ list have in common? And what about the healthy list?
- Have you heard of the term ‘ultra-processed’ food? What does that mean to you?

**5. Acceptability of marketing (30 mins)**

Now, thinking back to our earlier discussion of ads, I have a few to show you. As I show them to you, I want you to write down your initial thoughts of the ads – just write down whatever comes to mind and we will talk about it together.

[**SHOW AD, ALLOW TIME TO TAKE NOTES**, then go through questions below] Repeat this process for each ad; about 10 mins per ad, 3x examples]

| **Name of ad and link** | **Product** | **Company** | **Shown** |
| --- | --- | --- | --- |
| **KFC “Summer of Cricket”**  https://www.you  tube.com/watch  ?v=ipAXr2fbZps | Fried chicken, hot chips | KFC | This advertisement was shown over the summer of the 2017 Australian Cricket season, during non-child rated programs. |
| **Kellogg’s LCM snack, “Treehouse”**  https://www.you  tube.com/watch?  v=V6SK68nL_ZY | Puffed rice snack bars | Kellogg’s | This ad was shown during family viewing times during shows such as the Australian Open, the Big Bang Theory, and Married with Children. |
| **Coca Cola Energy, “Move with a new energy”**  <https://www.you>  tube.com/watch?  v=h0E8dyh1ifk | Energy drink | Coca-Cola | This ad is on the Coca-Cola Australia YouTube channel, and is being shown on non-child rated programs on free-to-air television. |

- What is the key message you get from this ad? How is it conveying this message to you?
- What audience do you think this ad is intended for? Where would you expect to see it? Why? [EXPLORE SOME KEY RESPONSES, THEN – what about if children saw this?]

Now, let me give you context for the ads.

[EXPLAIN LOOPHOLES] Children are exposed to lots of unhealthy food and drink marketing when they view programs that are not directed primarily at children. Older children, e.g. above 12, are also not included in any restrictions. Furthermore, food companies are allowed to market unhealthy foods and drinks using online means, and by sponsoring professional and local sports.

With that in mind, how does that make you feel about the ads? Any difference? Why? Why not?

(**PROBE** on: context? What do they think about context? Age appropriate? Key messages? Aspects of the ad e.g. characters portrayed, techniques used e.g. is it animated, etc. what if a child saw it on Facebook or Instagram?)

**6. What is the solution? (20 mins)**

Imagine that you are in charge of regulating marketing of food and drink like [INSERT UNHEALTHY EXAMPLE DISCUSSED]

- What do you think is important to keep in mind when we think about what should or shouldn’t be marketed? Or how it is marketed?
- Again, is it important to you that marketing and businesses do this in a responsible manner? Are they already being responsible? [**PROBE** in terms of marketing indirectly to children]
- Does anything need to change? If so, what and why?
- Tell me about what you think needs to happen when it comes to marketing of unhealthy food/drink. Why, why not? [**PROBE** in terms of marketing indirectly to children]
- How urgent is this, how important is this issue to you?

**Close and thank (5 mins)**

Is there anything else you would like researchers to know?

**Supplement 2: Main Focus Groups**

**Discussion guide for Main Focus Groups**

1. Intro (5 mins)

- Sign consent form, any questions

Today we are going to talk about your experiences watching and enjoying sport and the types of advertising you might come across, particularly major sponsors of some sports. There are no right or wrong answers. I’ll also let you know that we are being video- and audio recorded, and there are researchers sitting behind this mirror here. The ethics of market and social research dictate that if someone watching or listening to this group recognises one of you, they are obliged to leave the room. While I will ask for your names for our discussion, no one here will be explicitly named in this research.

2. Warm up (5 mins)

What sport do you enjoy watching, how do you watch it, and with who?

Tell me about your favourite/most memorable sporting moment?

3. Knowledge of sport sponsorship (10 mins)

You’re all here because you enjoy watching sport in some way. Whether it’s live or on tv, what does that experience mean to you? i.e. a way to relax, quality time with family/friends; why do you do it?

- Describe to me how you feel or what you associate with watching sport. Relaxed? Excited? Inspired? Part of a team/community? [write down associations on board]
- Besides the sport itself, what else do you associate with viewing a game/match? e.g. particular players, sounds, moods? NOTE any mention of junk/fast food
- What about the companies that sponsor the sports you watch? Can you name some? [write down sports and their brands]

4. Attitudes and opinions on sport sponsorship (20 mins)

Marketing is about “activities undertaken by a company to promote the buying or selling of a product or service” which can include sport sponsorship.

- In your opinion, is ‘sponsorship’ different to marketing’? [come to agreement on what we mean by sponsorship]
- Can you tell me some examples of what ways companies sponsor sport (e.g. logos on shirts, ads during TV, ads with sport stars etc)
- What do you think companies get out of sport sponsorship sport? (e.g. relationship with customers, reaching target audience, association of the brand with sport, image transfer)
- What are some examples of major sport sponsorships we’re aware of (e.g. brand/product and sport)
  - What do you think the links are between the sport and this [EXAMPLE] brand/product? Does this sponsorship make sense to you?
  - What do you associate with these brands when you see them while watching sport? E.g. Modern, patriotic, community, team spirit, etc.. How do they manage to make you feel this way? Does that fit in with the associations you mentioned earlier?
  - What do you think these brands want you to do when you see their sponsorship? Why do you think they’ve decided to sponsor this particular sport?
- Tell me about a sponsorship that makes the most sense to you. Why? And the one that makes the least sense? [NOTE mentions of gambling, alcohol, and probe on their reasons why]
  - Pretend that you are the head of sponsorship for your favourite sport. Write down what brands you would like to be aligned with the sport and what products or brands would not be okay
- Should the products be aligned with the sport in some way? E.g. sportswear vs. banks. Why, why not? Let’s go through our list and see what we think.
  - Do the brands align with the feelings you associate with watching the sport? Are there any that don’t make sense
  - [Focus on some examples of unhealthy products and medium of sponsorship – is it shirts? On the field?]
  - [PROBE on their opinions of prominent sportspeople promoting products – is this different to the sport as a whole being associated with certain products? How? Why?]
- Who is the target audience? Who are you watching with? What about kids?
- Other focus groups we’ve run tell us that some people aren’t very concerned about advertising of things that aren’t particularly ‘good’ for us, like fast food or soft drinks, because we all make our own choices. Thinking about sponsorship in sport, how concerned or not are you about what is advertised? Does it matter, given that we are a free market?
  - What are other things that sponsor sport that might be thought of as ‘bad for you’? What are the ‘good’ things, or the things you wouldn’t be concerned about at all?
  - Do you think that children should be free to attend or watch weekend sports without seeing advertising for unhealthy foods/things that aren’t so good for them? Should we do more to help reduce the exposure of unhealthy food to children through sport? Why/why not?
- What do you think is worst, out of alcohol, gambling and food sponsorship in sport? (Rank)

[PROBE on whether the reason for/approach to limiting one kind of sponsorship could also be applied to other types of sponsorship, e.g. limiting gambling promotion could lead to limiting KFC promotion]

- If limiting one kind of sponsorship was limited (e.g. gambling) what if it was replaced with fast food advertising? Would that be okay? Why or why not? [Compensation angle]

5. If we wanted to do something about this, what would we do? (15 mins)

- How important is it to you that sponsorship in sport through unhealthy products (like fast food, gambling, etc.) be addressed? If not, what (in the context of exposure to unhealthy products) is more important? Is it important at all? Why?

[IF IMPORTANT?] Let’s think about some ways to reduce exposure to junk/fast foods in sport.

[Write down what options they put up. Suggest others that were taken from earlier groups and in the literature – 1] tax breaks for healthier companies, 2] only allow non-food brands or healthier food brands 3] buy out with obesity prevention/healthy lifestyle campaigns 4] ban all alcohol and gambling products 5] ban companies with predominantly unhealthy portfolios 6] ban all products that don’t meet WHO nutrient criteria]

[If someone brings up buy out of tobacco – explain what they did: 1] restrict national sports events from having sponsors (not international) 2] allowing existing contracts to run out and no new ones to be signed 3] eventually restrict international sporting events 4] buy out from VicHealth 5] tobacco companies still allowed to give money to sports as long as they don’t receive any promotional or advertising benefits) Quit program became major sponsor of soccer, buy out by government

- Which ones do you support? Why or why not? Pros and cons? Rank in order of preference.
- What if some of these solutions made the cost of going to a game more expensive?
- Who would be responsible for enacting these changes?

6. Does anyone feel differently about brands sponsoring elite sport compared to at the start of our discussion? (5 mins)

Is there anything else you would like us to take back to the researchers?

**Supplement 3: Participant description of Main Focus Groups**

| **Participant number** | **Gender** | **Age (years)** | **Low or high SEP*** | **CALD*** | **Language other than English spoken at home?** | **Type and frequency of viewing live or televised sport*** | **How sport is viewed** |
| --- | --- | --- | --- | --- | --- | --- | --- |
| **Focus group 1: Parents of children, location central Sydney** | | | | | | | |
| FG1, P1 | F | 46 | H | N | N | Cricket, Tennis, Rugby League, Olympics | Free-to-air TV, Streaming devices, Live |
| FG1, P2 | M | 41 | H | Y | Y and English spoken | Cricket, Tennis, AFL, Rugby Union, Rugby League, Football, Olympics | Free-to-air TV, Streaming devices |
| FG1, P3 | M | 39 | L | Y | N | Cricket, Tennis, AFL, Rugby Union, Rugby League, Football, Olympics | Free-to-air TV, Streaming devices, Live |
| FG1, P4 | M | 39 | H | N | N | Cricket, Tennis, AFL, Rugby Union, Rugby League, Football, Olympics | Free-to-air TV, Streaming devices, Live, Foxtel |
| FG1, P5 | M | 43 | L | N | N | Tennis, Rugby League, Football | Free-to-air TV, Live |
| FG1, P6 | F | 33 | L | Y | Y and English spoken | Cricket, Tennis, AFL, Rugby Union, Rugby League, Football, Olympics | Free-to-air TV |
| FG1, P7 | F | 43 | H | Y | N | Olympics, Tennis | Free-to-air TV, Live |
| **Focus group 2: Parents of adolescents, location central Sydney** | | | | | | | |
| FG2, P1 | F | 38 | H | Y | N | Cricket, Tennis, AFL, Rugby Union, Rugby League, Football, Olympics | Free-to-air TV, Streaming devices, Live |
| FG2, P2 | M | 55 | H | Y | N | Cricket, Tennis, AFL, Rugby League, Olympics | Free-to-air TV, Streaming devices |
| FG2, P3 | M | 62 | L | Y | N | Cricket, Tennis, AFL, Rugby Union, Rugby Union, Rugby League, Football, Football | Free-to-air TV, Streaming devices, Live |
| FG2, P4 | F | 45 | H | Y | Y | Football, Cricket, Cricket, AFL, Rugby League, Olympics | Free-to-air TV, Streaming devices, Live |
| FG2, P5 | M | 30 | L | N | N | Cricket, Tennis, AFL, Rugby Union, Rugby League, Football, Olympics | Free-to-air TV, Streaming devices, Live |
| FG2, P6 | M | 49 | L | Y | N | Cricket, AFL, Rugby Union, Rugby League, Football, Olympics | Free-to-air TV, Streaming devices, Live |
| FG2, P7 | F | 54 | L | Y | N | Cricket, AFL, Rugby Union, Rugby League, Tennis, Football, Olympics | Free-to-air TV, Streaming devices, Live |
| FG2, P8 | F | 56 | H | N | N | Cricket, Tennis, Rugby Union, Football, Olympics, Rugby League | Free-to-air TV, Live |
| **Focus group 3: No children or adolescents, location central Sydney** | | | | | | | |
| FG3, P1 | F | 59 | H | N | N | Cricket, Tennis, AFL, Rugby Union, Rugby League, Football, Olympics | Free-to-air TV, Live, Public |
| FG3, P2 | F | 27 | H | Y | Y | Cricket, AFL, Rugby League, Football, Tennis, Rugby Union, Olympics | Free-to-air TV, Streaming devices, Live |
| FG3, P3 | M | 58 | L | N | N | Olympics, Tennis, Rugby Union, Rugby League, Football, Olympics | Streaming devices, Live, Foxtel |
| FG3, P4 | M | 52 | L | Y | N | Cricket, Tennis, AFL, Rugby Union, Rugby League, Football | Streaming devices, Live |
| FG3, P5 | F | 40 | L | N | N | Cricket, Tennis, AFL, Rugby Union, Rugby League, Football, Olympics | Free-to-air TV, Streaming devices, Live |
| FG3, P6 | M | 28 | H | Y | Y | Cricket, Tennis, Rugby Union, Rugby League, Football | Free-to-air TV, Streaming devices, Live, Internet Streaming |
| FG3, P7 | F | 67 | H | N | N | Cricket, Tennis, AFL, Rugby Union | Free-to-air TV |
| **Focus group 4: Parents of children, outer suburbs Sydney** | | | | | | | |
| FG4, P1 | F | 46 | H | N | N | Tennis, AFL, Rugby League, Football, Olympics | Free-to-air TV, Live |
| FG4, P2 | M | 37 | H | Y | Y | Cricket, Tennis, Tennis, AFL, Rugby Union, Rugby League, Football, Olympics | Live, Streaming devices, Live |
| FG4, P3 | M | 35 | H | N | N | Cricket, Tennis, AFL, Rugby Union, Rugby League, Football, Olympics | Free-to-air TV, Streaming devices, Live |
| FG4, P4 | M | 42 | H | Y | Y | Cricket, Tennis, Football, Olympics | Free-to-air TV, Streaming devices, Live |
| FG4, P5 | F | 32 | L | Y | N | Tennis, AFL, Rugby Union, Rugby League, Football, Olympics | Free-to-air TV, Streaming devices, Live |
| FG4, P6 | M | 46 | L | Y | Y | Cricket, Tennis, Olympics | Free-to-air TV, Streaming devices, Live |
| FG4, P7 | F | 38 | L | N | N | Cricket, Tennis, AFL, Rugby Union, Rugby League, Football, Olympics, | Free-to-air TV |
| *CALD - Culturally and linguistically diverse (Yes- speak a language other than English at home); SEP - Socioeconomic position (low - living in the top 50% of ranked postcodes in Australia according Socio-Economic Indexes For Areas, Index of Relative Socio-economic Disadvantage SEIFA IRSD, high – living in the bottom 50% of SEIFA IRSD)AFL - Australian Football League (known as "Football in Australia); Football - known as "Soccer" in Australia | | | | | | | |

**Supplement 4: Illustrative quotes from Main Focus Groups**

| **Theme** | **Sub-theme** | **Illustrative quotes** |
| --- | --- | --- |
| Sporting experiences | Personal experience | N/A |
| Ubiquity and pervasiveness of marketing | Increased marketing integration and effectiveness | *I guess they’re exposed to it, whether it’s sport or elsewhere, like toys on cartoon shows… FG4, P7,*  *What’s on the media, I mean, social media and stuff like that. In the news and stuff like that, we’re just made a lot more aware of it than what we probably were back 15-20 years ago. FG3, P4*  *Well, I don’t know, impact their consumer choices because I mean we all know that KFC’s, I mean it’s okay to eat it sometimes but you shouldn't be eating it all the time. And I’m saying they’re saying that, but I think there is a responsibility with some brandings or issues, like I don’t think gambling should be on the TV. FG4, P3*  *Yeah, there's no ads, per se, but they can run around with XXXX on their jerseys which, I guess, is not too much in your face. You kind of see it and you just kind of ignore it. Whereas, an ad is different. To me, the worst one is the Bet365 and the Ladbrokes – at the start of the game, before the game and then, at the end, after he's told you all the odds, he says, Gamble responsibly. Thanks, mate. FG2, P6*  *“Uber Eats was pretty repetitive, and they were done well, so you probably took that in your stride, I think.”* FG1, P4 |
| Role of unhealthy commodities in sport | Commercial benefit of sponsorship | *It’s exposure. It’s brand awareness. FG3, P4* |
|  | Effectiveness of marketing and sponsorship | *That’s all it is and it’s always in your face and it will influence the people watching it and that’s why they stopped cigarette advertising and cigarette sponsorship. FG2, p8*  *The target market [of advertising] is probably more the lower socio-economic part of society because they tend to smoke more, they tend to drink more, sadly, and they tend to gamble more. FG3, P3*  *…it [sport sponsorship] has a negative effect on the kids, so they are the ones who actually, when you’re an adult you can make your own decisions, but that’s the age, the vulnerable age where you’re actually showing them all that, and associating their heroes with those products, which they themselves would not be having them, the heroes themselves are not having that food or that drink, but the kids are associating themselves with that hero.” FG4, P6*  *Yeah, …definitely the betting. And even, when we’ve even watched it, I know as a family, and it pops up on the screen, that particularly my teenager will comment and say, “What’s that all…? …But it’s too young for them to process it all, and we didn’t have any of that when we were growing up with it all on the telly. FG4, P1*  *There is a high concentration, particularly in NRL, of beer and gambling. And even as an adult, you can tell that it’s designed to get you interested and you probably think what affect it might have on younger people. FG2, P3* |
|  | Contradictions of unhealthy commodities and sport | *…when you think of sport you think of being active, and you think of healthiness and… so then you don’t think that fast food is being healthy, or being active. FG4, P5*  *I don’t think there’d be too many of the cricket players eating KFC. FG1, P1*  *I think it can detract, some of the sponsorships can detract as well at the time when you’re watching the game live at home. FG4, P1*  *Gambling’s in a whole class of its own. Gambling corrupts sport … It’s a slightly different argument with fast food and alcohol. FG2, P2*  *Well, because they’re not healthy, but because the way, I guess marketing or sponsorship works that you associate the good things about NRL, and it has a halo effect on the brands associated with the [sport]… FG4, P3*  *I've got gambling as the most worst. The alcohol and then fast food. I'd see the gambling and alcohol as being a little bit more addictive and hard to kick than maybe the food. FG2, P1*  *I think as far as betting goes, I think that’s something that concerns me. FG4, P5*  *Betting obviously, and Star for that matter, is an extreme; you don’t want it. But for example, KFC, now, even kids get hooked up to, not necessarily to their hero, but then they say, “There’s some chicken, or there’s some fish there,” so I think it’s more, there’s virtually no way to stop kids from eating that. But as a family… FG4, P4*  *Anything that’s addictive … is detrimental to that sport.. FG2, P2*  *Yeah, definitely the betting. And even, when we’ve even watched it, I know as a family, and it pops up on the screen, that particularly my teenager will comment and say, “What’s that all…?” like this is probably going back a year or so ago, “What’s that all about?” So then we’ve explained it. But it’s too young for them to process it all, and we didn’t have any of that when we were growing up with it all on the telly. I think it can detract, some of the sponsorships can detract as well at the time when you’re watching the game live at home… FG4, P1* |
|  | Unhealthy habits associated with viewing sport | *I can't recall the last time I made a home-cooked meal when I was watching a game. FG2, P1*  *Yeah, as a viewer, we would be sitting down and having a McDonalds or a Coca Cola... FG3, P1*  *…definitely, you need beer to watch sport… FG1, P2*  *“Yeah, it’s all there but the simple fact is, before you arrive, if I go to watch it live in a stadium, the first stop is at the bar anyway. If I'm watching it with a bunch of mates in a pub, I'm drinking anyway. I didn’t need the ad”. FG2, P6* |
| Perceptions and opinions on restricting sports sponsorship and sport-related marketing | Mixed support | *Gambling’s in a whole class of its own. Gambling corrupts sport … It’s a slightly different argument with fast food and alcohol. FG2, P2*  *There is a high concentration, particularly in NRL, of beer and gambling. And even as an adult, you can tell that it’s designed to get you interested and you probably think what affect it might have on younger people. FG2, P3*  *If they limit by time during which you can advertise or something, I think that would probably be welcomed by most of the public. FG3, W1*  *I would say probably government should have the criteria of the advertisement quota for companies. I know it doesn’t make sense but still companies who are big names, who can spend millions of amount of money, they can be everywhere. Instead of that, they should have the, only one or two companies can sponsor a certain amount of, yeah, kind of thing. I’ve seen the IPL, I know, a few of the business men, they are chasing…. I know they have like 60 or 80 million to spend on, so those kind of things can restrict you FG3, P4*  *You can balance your diet, but one cigarette can cause you damage. FG1, P5* |
|  | Reduce exposure over complete restrictions | *I think having some limits is reasonable, maybe not an all-out ban, but say X amount of fast food ads [per hour] or something like that. FG1, P6* |
|  | Survival of sport | *…if they [the government] start with all these regulations, they could keep going and going and going. FG3, P2*  *I think having some limits is reasonable, maybe not an all-out ban, but say X amount of fast food ads [per hour] or something like that. FG1, P6* |
| Rights and responsibilities | Joint responsibility | *I think the government has a responsibility for anything that’s addictive or unhealthy to put laws in place to restrict as much as possible, how they're promoted.FG2, P2*  *I think we are quite firm in our minds that we don’t enjoy fast food, we don’t enjoy gambling, so it’s not so much for us but more the broader societal impact of it. Ideally, that would be restricted. Whether or not government can actually implement those restrictions without going too far is another question. FG3, P2*  *What they [sporting organisations] want to represent, what they want to build for the future of the Australian community. FG2, P1*  *It probably is on the sport really because they’re the ones selecting their sponsors, and I guess they have the right to say no and choose a different sponsor. FG3, P5*  *It probably is on the sport really because they’re the ones selecting their sponsors, and I guess they have the right to say no and choose a different sponsor. FG3, P7*  *Realistically, what can be done? Probably not government intervention. I’m just thinking lobbying the tournaments or the sports or the teams themselves to say, Hey, if it’s possible for you, go for another sponsor rather than McDonalds. FG3, P2*  *It’s also quite risky if you're relying on those sort of sponsors because, at any time, it might get taken away from you. So if I'm sitting on one of the sporting associations, I wouldn’t want to be dependent on something – my risk register would have very, very high risk about relying on something that might get banned and we might be in big trouble, so you want to get yourself off them as early as possible. FG2, P2*  *They [companies] probably have a right to do it [engage in sport sponsorship and marketing], because otherwise we’ve got to be a real nanny state, sort of thing. But in saying that, that’s probably like a kick below the belt sort of thing, like it can be done but it’s not the right thing to do when you’ve got something that children look into and bow down to. FG4, P7* |
|  | Complementary approaches | *The few that are paying, once you put restrictions, it does still hit the pocket for them, for the government, so why just we want the government to do everything for us? As parents, what role are we playing in our own house, because some of those are not actually free to air, we are actually subscribing to those channels, or are we going out to take them for a game, outdoor games, to spend time with our kids? So we’ve got choices, but we go what works for us, we’re working every day so it’s up to us parents to balance it. FG1, P3* |
|  | Visible policy coherence | *Like Government’s role is supposed to be government and do it ethically and responsibly and they've got to be consistent. On one hand they’ll say, We’ll put taxes on junk food or we’ll put educational material in schools so the kids don’t grow up obese or whatever health issues. On the other hand, they give carte blanche to the industries we've just discussed which set up people for long-term problems. So they have credibility, they need consistency not just talk out of two sides of the mouth. FG2, P3*  *To me, it’s a bit contradictory. Like they allow … they provide healthy recommendations for schools on their tuckshops and their shops in the schools – canteens – but then they allow things like advertising for fast food in sports and that and kids watch sport as well so it’s a bit … FG2, P5*  *It’s a double standard. FG2, P3*  *And all that, you sit down in government and do a cost benefit* [of banning unhealthy sport sponsorship]*, you'd ban it straight away.* FG2, P2  *I’m not a fan of the nanny state, but I wouldn't mind if the government sat down and said, “You can’t have any advertising,” because…I think these things, they just don’t align with … in PE [Physical education] class they’re talking about, “Well, what are some good healthy eating choices?. FG4, P3*  *Like Government’s role is supposed to be government and do it ethically and responsibly and they've got to be consistent. On one hand they’ll say, We’ll put taxes on junk food or we’ll put educational material in schools so the kids don’t grow up obese or whatever health issues. On the other hand, they give carte blanche to the industries we've just discussed which set up people for long-term problems. So they have credibility, they need consistency not just talk out of two sides of the mouth. FG2, P3* |

1. Obesity Policy Coalition. Overbranded, Underprotected 2019 [Available from: <https://www.opc.org.au/what-we-do/overbranded-underprotected>.

2. World Cancer Research Fund International. Restrict food advertising and other forms of commercial promotion. NOURISHING framework2019.

3. Smith R, Kelly B, Yeatman H, Boyland E. Food Marketing Influences Children's Attitudes, Preferences and Consumption: A Systematic Critical Review. Nutrients. 2019;11(4).

4. Carah N, Meurk C, Males M, Brown J. Emerging social media ‘platform’ approaches to alcohol marketing: a comparative analysis of the activity of the top 20 Australian alcohol brands on Facebook (2012-2014). Critical Public Health. 2018;28(1):70-80.

5. Watson WL, Lau V, Wellard L, Hughes C, Chapman K. Advertising to children initiatives have not reduced unhealthy food advertising on Australian television. J Public Health (Oxf). 2017;39(4):787-92.

6. Berry NM, Carter P, Nolan R, Dal Grande E, Booth S. Public attitudes to government intervention to regulate food advertising, especially to children. Health promotion journal of Australia : official journal of Australian Association of Health Promotion Professionals. 2017;28(1):85-7.

7. Chambers T, Sassi F. Unhealthy sponsorship of sport. Bmj. 2019;367:l6718.

8. Flint SW, Peake R. Lead by example: should sport take a stand against brands of unhealthy consumption? Public Health. 2016;134:117-9.

9. Australian Bureau of Statistics. Socio-Economic Indexes for Areas. 2018.

10. Australian Bureau of Statistics. Australia's Population by Country of Birth. 2019.
